# Extracellular Acidosis Stimulates NHE2 Expression through Activation of Transcription Factor Egr-1 in the Intestinal Epithelial Cells

**DOI:** 10.1371/journal.pone.0082023

**Published:** 2013-12-23

**Authors:** Saminathan Muthusamy, Ming Cheng, Jong-Jin Jeong, Anoop Kumar, Pradeep K. Dudeja, Jaleh Malakooti

**Affiliations:** Division of Gastroenterology and Hepatology, Department of Medicine, University of Illinois at Chicago, Chicago, Illinois, United States of America; University of Kansas School of Medicine, United States of America

## Abstract

Na^+^/H^+^ exchangers (NHEs) play important roles in regulating internal pH (pH_i_), cell volume and neutral Na^+^ absorption in the human intestine. Earlier studies have shown that low extracellular pH (pH_e_) and metabolic acidosis increases the expression and function of NHE1-3 genes. However, transcriptional mechanisms involved remained unknown. Therefore, we investigated the molecular mechanisms underlying acid-induced NHE2 expression in C2BBe1 and SK-CO15 intestinal epithelial cells. Assessing total RNA and protein by RT-PCR and Western blot analysis, respectively, displayed significant increases in the NHE2 mRNA and protein levels in cells exposed to acidic media (pH 6.5 and 6.7) compared to normal medium. Acid treatment was also associated with a significant enhancement in NHE2 transport activity. Quantification of the heterogeneous nuclear RNA indicated that the rate of NHE2 transcription was increased in response to acid. Furthermore, acid caused a significant increase in NHE2 promoter activity confirming transcriptional upregulation. Through functional and mutational studies the acid-response element was mapped to a 15-nucleotide GC-rich sequence at bp −337 to −323 upstream from the transcription start site. We previously identified this element as an overlapping Egr-1/Sp1/Egr-1 motif that was essential for the NHE2 upregulation by mitogen-induced transcription factor Egr-1. Cells exposed to acid exhibited a temporal increase in Egr-1 mRNA and protein expression. These events were followed by Egr-1 nuclear accumulation, as detected by immunofluorescence microscopy, and potentiated its *in vitro* and *in vivo* interaction with the NHE2 promoter. Disruption of ESE motif and knockdown of Egr-1 expression by targeted small interfering RNA abrogated the acid-induced NHE2 transcriptional activity. These data indicate that the acid-dependent NHE2 stimulation is implemented by transcriptional upregulation of NHE2 via acid-induced Egr-1 in the intestinal epithelial cells.

## Introduction

During extracellular acidosis protons passively diffuse into cells resulting in a concomitant decrease in intracellular pH (pH_i_). Although the buffering capacity of the cells counteracts the small perturbations in the physiological pH, pH changes beyond that necessitate re-establishment of the pH_i_ homeostasis through activation of the pH-regulating systems. In this regard, multiple transport systems are involved in controlling the pH_i_ balance by extruding H^+^ out of the cell. The Na^+^/H^+^ exchanger (NHE) system plays a major role in this process in mammalian cells [1], [2]. NHEs catalyze the electroneutral exchange of an intracellular H^+^ for an extracellular Na^+^, thereby eliminating excess acid. The NHE family is composed of 10 isoforms. These isoforms are expressed in a ubiquitous or a cell- and tissue-specific manner. The sub-cellular localization of these isoforms also varies. In polarized epithelial cells, they are located in different sub-domains of the plasma membrane or they are found in the intracellular organelle membranes [3]–[5]. The intestinal epithelial cells express NHE2, NHE3, and NHE8 on their apical and NHE1 and NHE4 on their basolateral membrane [3], [6], [7]. NHE1, the first isoform identified, is primarily involved in the regulation of pH_i_ and cell volume. NHE2 and NHE3 are involved in transepithelial Na^+^ absorption and also participate in regulation of pH_i_ homeostasis and maintenance of cell volume [4], [6].

Previous studies have shown that NHE activity is stimulated by lowered pH_i_, which may be caused by a fall in pH_e_ or cellular metabolic activities [8]. In this regard, long-term incubation of various renal cells in low pH media or chronic metabolic acidosis *in vivo* in animal models increased the NHE1 and NHE3 expression and activity [9]–[12]. A similar effect of metabolic acidosis on the NHE2 and NHE3 expression and activity was reported in the colonic mucosa from rats fed with NH_4_Cl to induce acidosis, where the increases in NHE2 and NHE3 mRNA and protein levels were associated with enhanced sodium absorption [13]. However, the molecular mechanisms underlying the effects of acidosis on the expression of NHEs are not known. In this study, we sought to determine the effect of extracellular acidification on NHE2 expression and activity by exposing the human intestinal epithelial cells to acidic media and to identify the *cis-* and *trans-acting* factors that play a role in mediating the effects of acid on NHE2 expression. Our findings support and extend the previous data and indicate that activation of acid-induced transcription factor Egr-1 is responsible for the transcriptional upregulation of NHE2 in response to acid in intestinal epithelial cells.

## Materials and Methods

### Plasmids

The NHE2 promoter constructs and site-directed mutagenesis were as in [14] except that sequences between bp +150 and the ATG, translation initiation site, were deleted as described in [15].

### Cell culture and transfection

C2BBe1 cell line was purchased from American Type Culture Collection (ATCC) (Rockville MD) and maintained as described previously [14]. SK-CO15, a human colonic adenocarcinoma cell line [16] was maintained in DMEM containing 10% FBS, 100 units/ml penicillin and 100 µg/ml streptomycin. For acid treatment DMEM medium containing 25 mM HEPES was used and the culture medium was adjusted to pH 6.5 or 6.7 by the addition of 1 N HCl as described previously [17]. Cells were incubated at 37°C in a 5% CO_2_ humidified incubator and pH was monitored at each time point and remained unaltered throughout completion of the experiment. Cell viability was assessed using the Trypan Blue dye exclusion (Sigma-Aldrich, St. Louis, MO) method with no significant loss of cell viability after 24 h of acid exposure. Cells were transfected with plasmids using LipoFectamine 2000 (Invitrogen). Transfected cells were serum-starved (20 h) and exposed to acidic media (pH 6.5 or 6.7) for 20–24 h. Forty-eight hours post-transfection, cells were washed, collected, and lysed in passive lysis buffer (Promega, Madison WI). Protein concentrations were determined using Bradford Assay (Bio-Rad Laboratories, Hercules, CA). Luciferase activities were measured using GLOMAX Luminometer (Promega) and normalized to total cell proteins [14]. For small interfering RNA (siRNA) transfection, cells were transfected with Egr-1 specific siRNA or control non-targeting siRNA (100 nM each) (Santa Cruz Biotechnology, Santa Cruz CA), after 24 h transfected again with p-415/+150, followed by exposure to acid (20 h), and luciferase activities determined [15]. All transfections were performed in triplicates and repeated at least three times.

### ^22^Na^+^-uptake assay

Cells were seeded at a density of 5×10^4^ cells/well in 24-well plastic plates and Na^+^/H^+^ exchange activity was determined 14 days post-plating. The control and acid (pH 6.5, 24 h) preincubated cells were washed with 1×PBS and incubated in Na^+^-free acid load solution consisting of (in mM) 50 NH_4_Cl, 70 choline chloride, 5 KCl, 1 MgCl_2_, 2 CaCl_2_, 5 glucose, and 15 MOPS (pH 7.0) at room temperature for 30 minutes. The cells were then subjected to two rapid washes with a solution containing (in mM) 120 choline chloride and 15 Tris-HEPES (pH 7.5). The wash solution was removed and replaced with uptake buffer containing (in mM) 10 NaCl, 110 choline chloride, 1 MgCl_2_, 2 CaCl_2_, and 20 HEPES (pH 7.4) and 1 µCi/ml of ^22^Na (New England Nuclear Life Science Products, Boston, MA), with or without HOE-694 (NHE2-specific inhibitor at 50 µM) and processed as described previously [15].

### RNA preparation and semi-quantitative RT-PCR

Total RNA was extracted and treated with DNase I using RNeasy Kit (Qiagen Inc. Valencia, CA). Three µg of RNA was reverse transcribed as previously described [15]. One-tenth of the cDNA was utilized for PCR reactions. NHE2 mRNA amplifications were performed with both oligo-(dT) and random hexamer-primed cDNA. The NHE2 heterogeneous nuclear RNA (hnRNA) was amplified using cDNA produced by random hexamers as a template and a forward primer from exon-5 and the reverse primer from intron-5. Primer sequences are presented in Table S1. The PCR conditions were denaturation at 95°C for 30 sec, annealing at 58°C for 30 sec, and extension at 72°C for 60 sec for 35 cycles with an initial denaturation at 95°C for 5 min. All PCR data were normalized against glyceraldehyde 3-phosphate dehydrogenase (GAPDH) gene mRNA expression, which was amplified for 20 cycles with the same parameters as above.

### Western immunoblot analysis

Western blotting was carried out as previously described [15]. The NHE2 antibody was a gift from Dr. Mark Musch (University of Chicago, Chicago IL) [18] and is a mouse monoclonal antibody. After electrophoresis the proteins were blotted onto a PVDF membrane (Millipore). The blotted membrane was blocked in TBST containing 5% nonfat milk for 1 h at room temperature. The NHE2 antibody was diluted (1∶1000) in TBST containing 5% nonfat milk and the blot was incubated at 4°C overnight in a rotary shaker. Membrane was washed 3 times with TBST 7 min each and incubated with anti-mouse IgG-HRP (1∶10000) in TBST containing 5% nonfat milk for 1 h at room temperature and washed 3 times in TBST and the blots were imaged with Western Lightning ECL Pro (PerkinElmer). Egr-1 antibody was from Santa Cruz Biotechnology (Cat #sc-110). Actin and GAPDH antibodies were from Sigma. The band density analyses were performed by densitometry scanning using the ImageJ software (http://rsbweb.nih.gov/ij/).

### Immunofluorescence microscopy

Cells were grown on glass coverslips to ∼90% confluence. After serum-starvation and acid treatment (60 and 90 min), cells were fixed with 1% (v/v) paraformaldehyde in phosphate-buffered saline (PBS) for 60 min at ambient temperature and stained as described previously [19]. Briefly, the fixed cells were washed, incubated in blocking buffer (1× PBS/3% normal goat serum/0.05% saponin) (30 min), and hybridized with Egr-1 antibody (Santa Cruz) (1∶100) (1 h) at room temperature. Then coverslips were washed 3-times with 0.05% saponin/PBS solution and incubated with Alexa Fluor 488-conjugated anti-rabbit secondary antibody (Invitrogen) (1∶300) for 60 min at room temperature. The nuclei were stained with DAPI (100 ng/ml) in 1× PBS for 2 min. The coverslips were mounted on slides using ProLong antifade reagent (Invitrogen) and allowed to dry. Cell Images were captured with a Leica DM4000B epifluorescence microscope and digital images were prepared with Slide Book software (Intelligent Imaging Innovations Inc. Colorado, USA).

### Nuclear extract preparation and Gel Mobility Shift Assay (GMSA)

Nuclear extract preparations were carried out using NucBuster Extraction Kit (Calbiochem). DNA-Protein binding reactions were performed as described in [14]. SP1, SP3, and Egr-1 antibodies were from (Santa Cruz Biotechnology).

### Chromatin Immunoprecipitation (ChIP) assay

ChIP assay was performed on C2BBe1 control and acid treated cells using the EZ-ChIP Assay Kit (EMD Millipore, USA) according to the manufacturer protocol. DNA-protein complexes were immunoprecipitated using an anti-Egr-1 antibody. The purified co-immunoprecipitated chromatin was used as a template for PCR amplification using the forward and reverse primers (Table S1) that start at bp −514, and −317 upstream from the NHE2 transcription initiation site, respectively. This primer set amplifies the NHE2 promoter region harboring the Egr-1/Sp1/Egr-1 (ESE), acid-response element. All PCR products were resolved on a 1.5–2% agarose gel, stained with ethidium bromide and documented using the gel documentation system Alpha Imager 1220 (Alpha Innotech Corporation, USA).

### Statistical analysis

Data are presented as mean ± SEM. The difference between two groups was evaluated by Student's *t-test*. *P* value ≤0.05 was considered significant compared to control. All experiments were repeated at least 3 times for statistical analysis.

## Results

### Exposure to acidic media enhances the human NHE2 mRNA and protein expression in the intestinal epithelial cells

**Figure 1:**
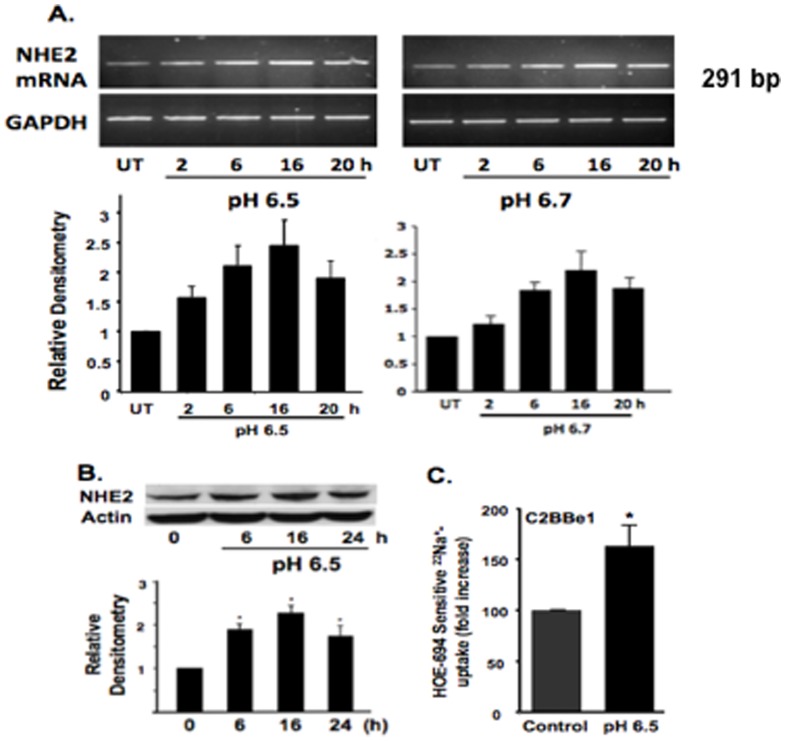
Acid exposure induces the NHE2 mRNA and protein levels and increases its transport activity in C2BBe1 cells. A) Time-dependent induction of NHE2 mRNA by acid. Four days post-plated confluent cells were cultured in control or acidic media (pH 6.5 and 6.7) as indicated and NHE2 mRNA levels determined by RT-PCR. B) Western blot of cell extracts probed for NHE2 and actin (loading control), also showed acid and time-dependent increase in NHE2 protein levels. Densitometry analyses show the relative abundance of NHE2 mRNA (A) or protein (B) with respect to control. C) Acid increases the NHE2 functional activity. NHE2 activity was determined in the presence of NHE2 inhibitor HOE-694 (50 µM) as described in Materials and Methods. The control is set arbitrarily to 100% and increases in NHE2 activity is presented as percent of control. Values are means ± SEM, n≥6. * p<0.05.

To evaluate the effect of acidic environment on the human NHE2 mRNA expression in intestinal epithelial cells, C2BBe1 cells were exposed to low pH media (6.5 and 6.7) and NHE2 mRNA levels determined by RT-PCR. Compared with control cells incubated in normal media (pH 7.4), exposure to acid increased the NHE2 mRNA abundance by ∼2–3-fold in a time-dependent manner (Figure 1A). Similar levels of NHE2 upregulation were observed using quantitative real-time PCR (data not shown). Acid exposure also enhanced NHE2 protein abundance (Fig. 1B). The maximal increase in protein levels was observed at 16 h post-treatment. This increase in NHE2 protein levels was associated with acid-induced NHE2 mRNA upregulation, which preceded the enhanced protein accumulation. Next, the impact of acid on NHE2 transport activity was evaluated by ^22^Na^+^-uptake studies. Long-term acid treatment (24 h) to C2BBe1 (Fig. 1C) elicited a significant increase in NHE2-mediated transport activity as determined by acute NH_4_Cl prepulse in the presence or absence of the NHE2-specific inhibitor HOE-694 (50 µM). Together, these results demonstrated that low pH_e_ leads to increased NHE2 expression, which was associated with enhanced NHE2 activity. To establish that acid-induced stimulation of NHE2 was not specific to C2BBe1 cells, the human colonic adenocarcinoma cell line SK-CO15 was also subjected to similar treatments. As with C2BBe1 cells, acid exposure enhanced NHE2 mRNA and protein levels as well as transport activity in SK-CO15 cells (Fig. S1) indicating that the effect of acid on NHE2 expression is not cell line specific.

### Extracellular acidification stimulates the rate of NHE2 transcription

**Figure 2:**
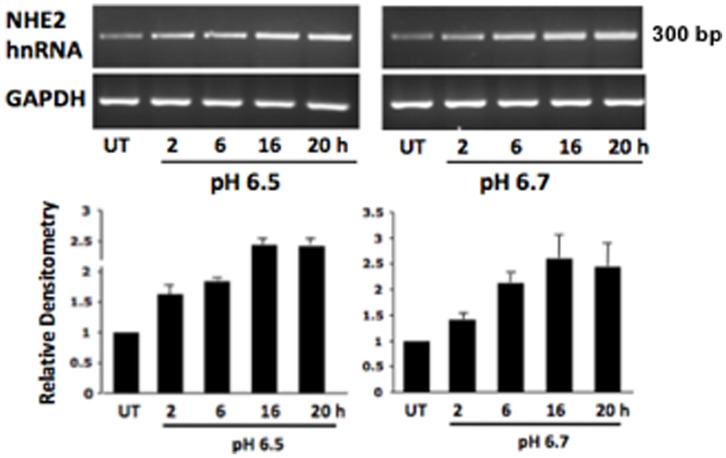
Acid exposure increases the NHE2 hnRNA levels. C2BBe1 cells were grown to confluence for four days and after serum-starvation treated or not with acid (pH 6.5 and 6.7) for different time intervals. The levels of hnRNA were analyzed by RT-PCR and quantified by densitometry scanning. N = 3, a representative gel is shown. Data are presented relative to control set arbitrarily to 1.

The acid-dependent increase in NHE2 mRNA levels may occur through stimulation of transcription rate or mRNA stability. Quantification of hnRNA provides a direct measure of the gene transcription rate [20]. Hence, to specifically target the NHE2 hnRNA, a primer set composed of a forward primer from exon-5 and a reverse primer from intron-5 was used to amplify the NHE2 hnRNA and quantify its abundance during acid incubation. Time-course analysis of acid-treated cells showed increased NHE2 hnRNA as early as 2 h and augmented hnRNA abundance in the following time points tested (Fig. 2). These increases in hnRNA levels correlated with the abundance of NHE2 mRNA in response to acid (Fig. 2A) suggesting that acid-induced NHE2 mRNA upregulation occurs through enhanced transcription rate.

### Extracellular acidification stimulates transcriptional activity of NHE2 promoter

**Figure 3:**
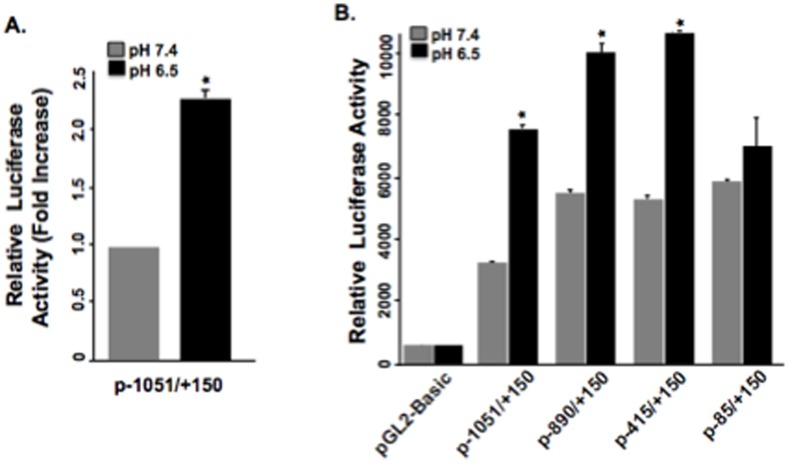
NHE2 promoter activity is upregulated by acid. A) One day post plated cells were transfected with the indicated NHE2 promoter constructs and incubated in control or acidic media (pH 6.5) for 20 h. Luciferase activities were determined and shown as average luciferase activity relative to control after normalization to total cell proteins. A) NHE2 promoter activity in response to acid treatment. B) Acid induces the NHE2 promoter activity through bp −415 to −85. * *p*<0.05, compared to the same construct grown in normal media.

In C2BBe1 cells transfected with NHE2 promoter-luciferase construct p-1051/+150, acid treatment (24 h) led to a significant increase in luciferase activity (∼2.2-fold) compared to control (Fig. 3A). These results confirmed that the effect of acid on upregulation of NHE2 expression is through transcriptional activation. The location of the potential acid-responsive cis-element(s) on the promoter was determined by 5′-deletion analysis of the p-1051/+150. A series of sequential 5′-truncated NHE2 promoter constructs were transiently transfected into C2BBe1 cells and reporter gene activities were determined. As shown in Figure 2B, similar promoter activities were observed in response to acid with deletions up to position −415. However, promoter activity was significantly decreased by deletion to bp −85, suggesting that the region between bp −415 to −85 harbors the acid response element(s). Various transcription factors including NF-κB, AP-1, and Egr-1 are activated in response to extracellular acidification in different cells [17], [21]–[24]. Interestingly, two Egr-1 binding sites are located in the NHE2 promoter between bp −415 to +1. Of these two, the distal binding site at position −337/−323, which is composed of overlapping Egr-1/Sp1/Egr-1 recognition sites (ESE), mediates the stimulatory effect of mitogenic ligand phorbol 12-myristate 13-acetate (PMA) on NHE2 promoter activation through Egr-1 binding [14].

### Acid-treatment augments Egr-1 expression, and induces its translocation to the nucleus

**Figure 4:**
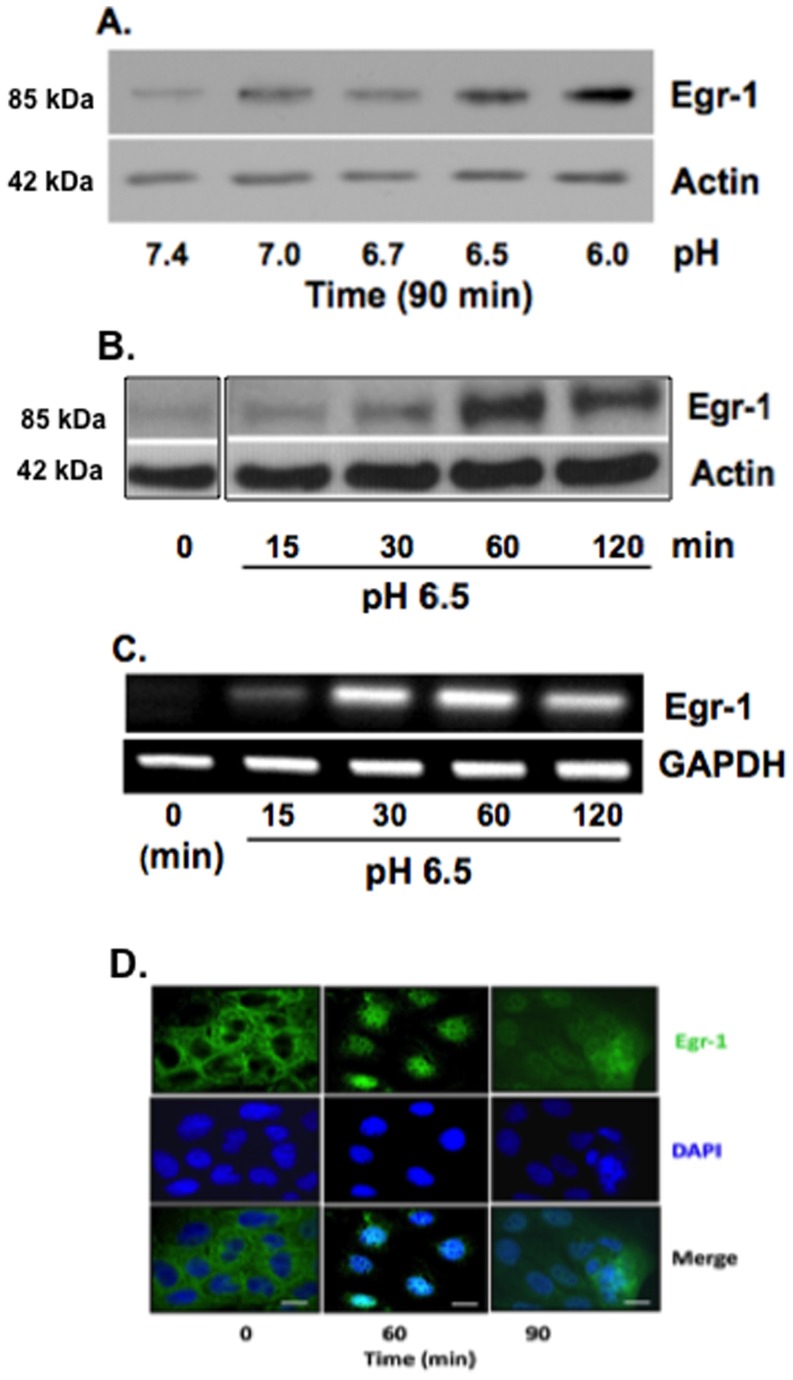
Acid induces Egr-1 expression and translocation to the nucleus in C2BBe1 cells. A) Four days post plated cells were exposed to media acidified to different pH for 90 min. Immunobloting was performed on whole cell lysates using Egr-1 antibody and β-actin as loading control. B) Time-course analysis of Egr-1 protein expression in cells exposed to acid (pH 6.5). C) RT-PCR analysis show pH- and time-dependent Egr-1 mRNA expression in response to acid. D) Acid exposure leads to Egr-1 translocation to the nucleus. Immunofluorescence microscopy of cells treated with acid and stained for Egr-1 (green) and nuclei (blue). Scale bars: 10 µm.

To assess the potential role of Egr-1 in acid-mediated NHE2 transcriptional activation, we investigated the effects of acid on Egr-1 expression. Acid treatment elicited a marked increase in Egr-1 protein levels in C2BBe1 cells in a pH- and time-dependent manner (Fig. 4A and B). The higher levels of Egr-1 protein correlated with decreasing pH values with maximal expression at pH 6.0. Furthermore, Egr-1 protein expression was transient with a peak at 60 min post-treatment and gradually decreased thereafter (data not shown). Exposure to acid also induced a marked and temporal increase in Egr-1 mRNA abundance in these cells (Fig. 4C). Egr-1 nuclear localization is necessary for its function as a transcription factor; therefore, we analyzed Egr-1 nuclear accumulation by immunofluorescence microscopy in acid-treated C2BBe1 cells. As shown in Figure 4D, after one hour of acid exposure the cytoplasmic Egr-1 is almost entirely found inside the nucleus.

### Acid exposure enhances Egr-1 interaction with the NHE2 promoter

**Figure 5:**
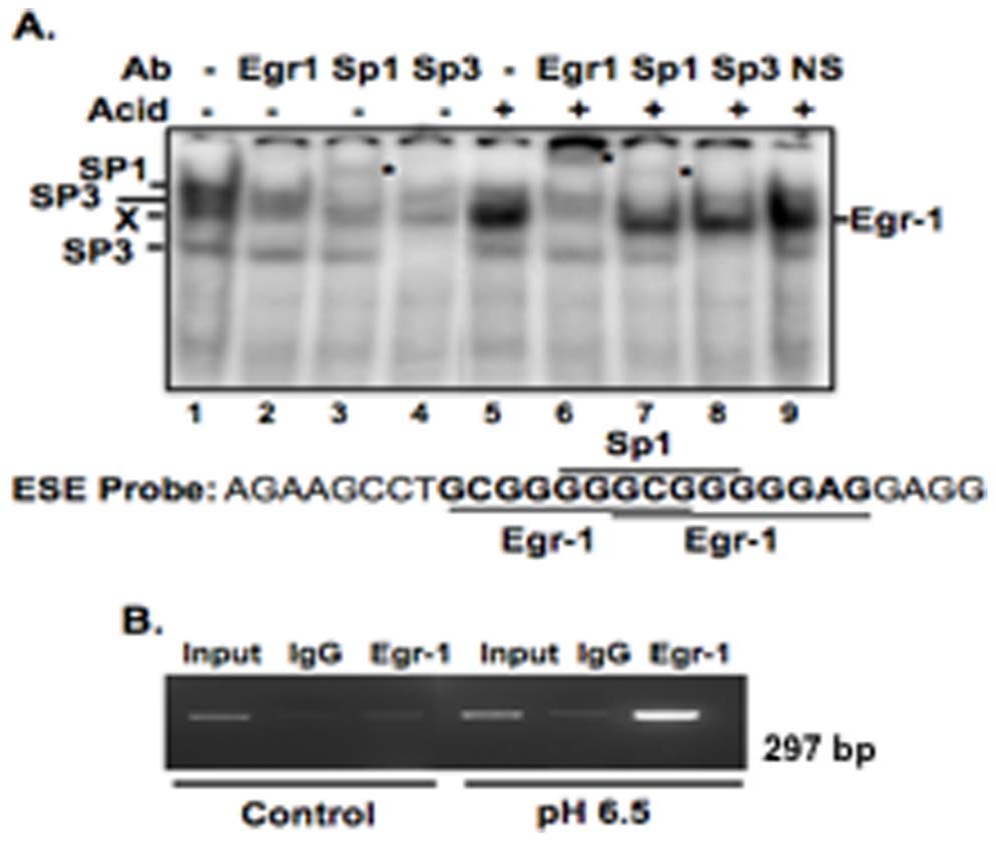
Acid-induced Egr-1 directly interacts with NHE2 promoter. A) In gel mobility shift assay. Acid promotes Egr-1 interaction with probe spanning the ESE motif. As shown previously [14] and confirmed here by supershift assays, in nuclear extracts (5 µg) from untreated cells SP1 and SP3 interact with the ESE probe (lanes 1–4). Acid induces formation of a major DNA-protein complex (lane 5), which is supershifted with anti-Egr-1 antibody (lane 6), but not with SP1, SP3, and nonspecific antibodies (lanes 7–9). * Denotes supershifted bands. B) ChIP assay. Chromatin-protein complexes were immunoprecipitated with Egr-1 or normal rabbit IgG and amplified using NHE2 specific primers (Table S1).

To examine whether a correlation exists between the acid-induced Egr-1 nuclear accumulation and NHE2 upregulation, we initially studied, *in vitro* and *in vivo* binding of Egr-1 to the NHE2 acid responsive region. As we have shown previously [14], using an oligonucleotide spanning the ESE motif as the end-labeled probe and nuclear proteins from untreated cells, transcription factors SP1/SP3 and an unidentified protein interacted with the probe (Fig. 5A, lanes 1–4), however acid treatment induced binding of a new prominent band with the same probe (Fig. 5A, lane 5). This major band was supershifted by an Egr-1 antibody (Fig. 5A, lane 6), but not with SP1, SP3 or non-specific antibodies (lanes 7–9). Further, since Egr-1 recognition site overlaps the SP1/SP3 binding site, binding of Egr-1 resulted in reduced levels of bound SP1/SP3 (Fig. 5A, lanes 5). ChIP analysis was used to examine the effect of acid on Egr-1 association with the NHE2 promoter *in vivo*. Cross-linked chromatin fragments were co-immunoprecipitated with Egr-1 antibody from cells treated with or without acid for 2 h and subjected to PCR. A markedly enriched level of chromatin containing the NHE2 promoter was observed in acid-treated cells compared to the respective control IgG or samples from untreated cells (Fig. 5B). These results confirmed increased accumulation of Egr-1 on the NHE2 promoter in response to acid and unequivocally demonstrated the direct interaction of Egr-1 with the NHE2 promoter.

### Acid-induced upregulation of NHE2 is mediated by Egr-1

**Figure 6:**
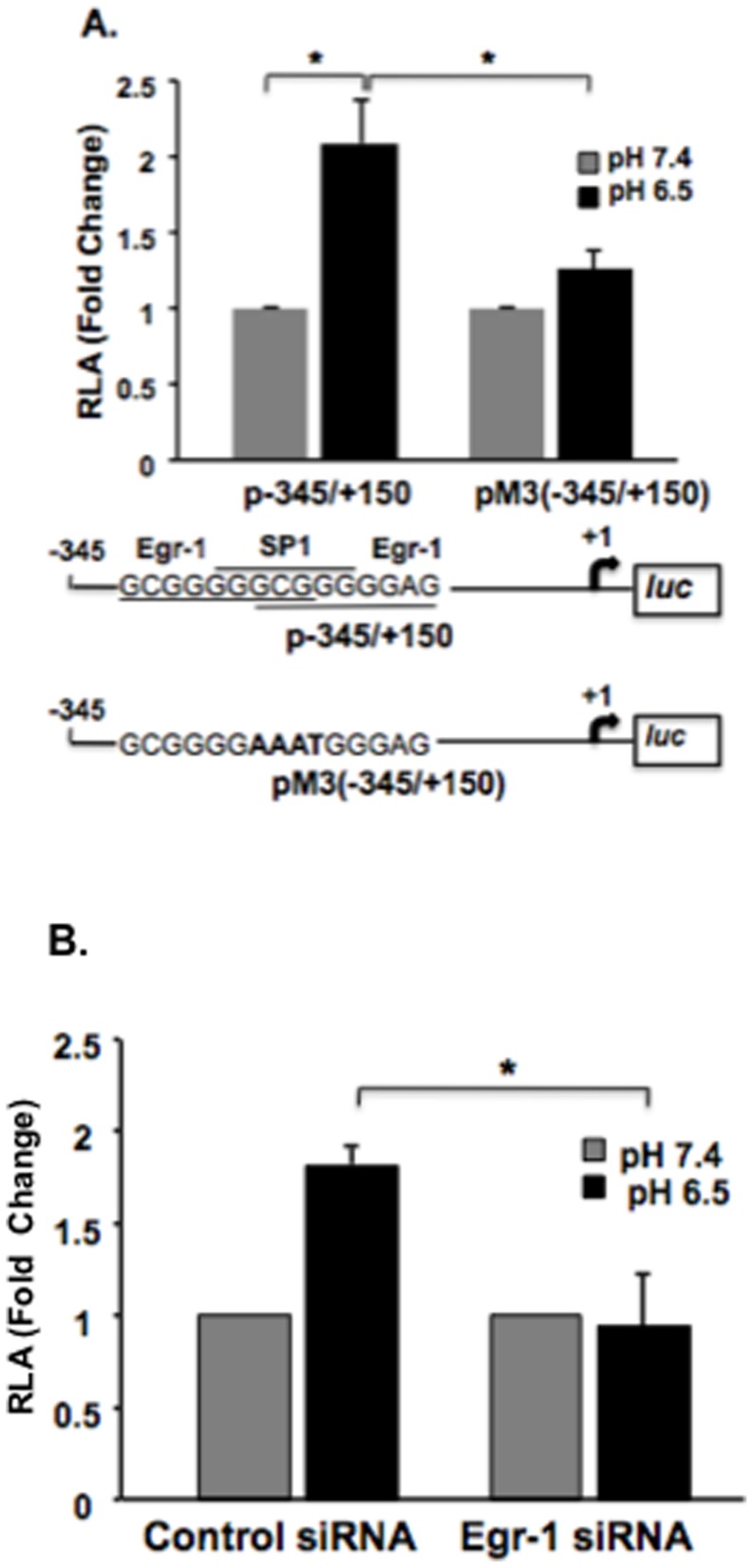
Egr-1 is necessary for the acid-induced upregulation of NHE2 promoter activity. A) Disruption of ESE motif abrogates acid-induced NHE2 promoter activity. One day post plated C2BBe1 cells were transfected with p-345/+15 or mutated pM3-345/+150 and the impact of ESE mutation on acid-induced NHE2 promoter activity was determined by luciferase assays. N = 3, * p<0.05. B) Silencing of Egr-1 by siRNA treatment blocks acid-induced NHE2 promoter activity. Cells were transfected with Egr-1 specific siRNA or control siRNA prior to transfection with p-415/+150. After acid treatment (pH 6.5, 20 h) cell lysates were prepared for luciferase assays. Fold change in luciferase activity was calculated from the respective untreated cells. N = 3,* *P*<0.05.

To assess to importance of ESE element on the modulation of NHE2 transcriptional activity by acid, base substitutions were introduced to ESE motif in p-345/+150 promoter construct. This plasmid harbors a promoter region immediately upstream from the ESE motif to position +150 downstream from the NHE2 transcription initiation site. In GMSA, these base substitutions prevent Egr-1 interaction with the ESE probe (data not shown). As shown in Figure 6A, disruption of ESE motif was sufficient to abrogate ∼80% of the increase in acid-induced NHE2 promoter activity in cells transfected with pM3-345/+150 compared to p-345/+150 indicating that an intact ESE element was essential for NHE2 regulation by acid. To further demonstrate the critical role of Egr-1 in the transcriptional up-regulation of NHE2 expression in response to acid, Egr-1 expression was silenced by Egr-1 targeted siRNA and then cells were transfected with p-415/+150 and subjected to acid treatment for 20 h. The acid-dependent NHE2 promoter activity was reduced to basal level in Egr-1 siRNA treated cells, whereas the non-targeting siRNA showed no effect (Fig. 6B). Together, these data indicated that acid-dependent stimulation of NHE2 promoter is attributable to activation of Egr-1.

## Discussion

Previously, we cloned the NHE2 cDNA from human normal colonic cDNA. By heterologous expression in the NHE deficient LAP-1 fibroblasts, we demonstrated that this cDNA encodes a functional transporter capable of promoting Na^+^-dependent pH_i_ recovery following an acid prepulse [25]. NHE2 is expressed throughout the intestinal tract; however, it is predominantly expressed in the colon and appears to play an important role in Na^+^ absorption as well as maintenance of pH_i_ homeostasis in the colon [26], [27]. In the current report, we sought to investigate whether extracellular acidification regulates the human NHE2 gene expression in cultured intestinal epithelial cells and to define the mechanisms involved.

Previously we reported that NHE2 promoter activity is upregulated via PMA-induced Egr-1 [14]; and demonstrated that Egr-1 acts as a mediator of PMA-activated PKCδ-ERK1/2 pathway connecting the upstream signals to the NHE2 upregulation [19]. The expression of Egr-1 in various cell types can be induced by different stimuli including growth factors, cytokines, cell injury, and various cellular stress conditions including acidosis [14], [17], [24], [28], [29]. Our current studies revealed that the cis-element mediating the PMA-response is also responsible for the acid-induced activation of NHE2. The NHE2 promoter-reporter gene analyses provided direct evidence that transcriptional mechanisms were involved in upregulation of NHE2 expression in response to acid through activation of Egr-1-dependent pathways. Acid exposure led to differential co-occupancy of ESE motif with SP1/SP3 and EGR-1 proteins where Egr-1 represented the majority of the bound nuclear proteins. This stimulus-specific mode of NHE2 regulation was also observed with PMA treatment of the C2BBe1 cells [14] and other genes regulated by the overlapping Sp1/Egr-1 motif on their promoter [30]–[32]. These data demonstrate dynamic and context-specific interactions of selected nuclear proteins with the NHE2 promoter as a mechanism for transcriptional regulation of the NHE2 gene and its consequent functional implication in the intestinal epithelial cells.

Studies by Gens, et al. [33] demonstrated that under acute acid-load conditions the abundance of NHE2 protein increases in the plasma membrane of transfected fibroblasts leading to elevated NHE2 activity. Here we have shown that long-term acid exposure also enhances the NHE2 activity. Therefore, it is plausible that acid-dependent increases in NHE2 protein levels may lead to its increased abundance in the plasma membrane and augment NHE2-dependent transport activity. With regards to the functional relevance of NHE2 activation by acid, high concentration of the short chain fatty acids (SCFAs) in the colon, which are produced by bacterial fermentation of undigested carbohydrates and proteins, generates an acidic niche in the lumen of proximal colon with pH values ranging from 6.2 to 6.6 in different species [34]–[36]. Nevertheless, the colonocytes maintain the pH_i_ at physiological range. In such cases, activation of the NHE system, including NHE2, is expected to lead to extrusion of H^+^ and maintenance of pH_i_ balance, enabling the cells to tolerate the external acidic conditions.

Recent studies have implicated NHE2 in tissue repair processes in mouse gastric epithelium [37], and intestine where the presence of NHE2 increased recovery from ischemia-related barrier malfunction [38]. In this regard, Egr-1 has been shown to impact cell injury and repair via activation of the target genes such as VEGF, bFGF, and PDGF [29]. Whether Egr-1 plays a role in the repair process via upregulation of NHE2 as well is not known and warrants further studies.

In conclusion, we provided evidence that *in vitro* exposure of the intestinal epithelial cells to acidic environment leads to increased NHE2 expression via transcriptional upregulation involving Egr-1. Acid exposure resulted in increased NHE2 mRNA and protein abundance and augmented the NHE2-mediated NHE activity. This was correlated with marked enhancement of Egr-1 mRNA and protein expression followed by its nuclear translocation and binding to NHE2 promoter. Our study, for the first time, identifies the ESE motif as a specific cis-element and Egr-1 as its cognate trans-acting regulatory factor contributing to NHE2 stimulation by extracellular acidification. Overall, NHE2 activation by acid appears to mimic, in part, the *in vivo* adaptation response to the metabolic acidosis thereby may contribute to protection against intracellular acidification.

## Supporting Information

Figure S1 Acid-induced upregulation of NHE2 mRNA (A), protein (B), and transport activity (C) in SK-CO15 intestinal epithelial cells. Cells were cultured in normal media to near confluence prior to acid treatment for RNA and protein preparations and for 14-days post-plating for ^22^Na^+^-uptake studies. For acid-treatment cells were incubated in serum-reduced media (0.1% FBS, 20 h) and then in serum-reduced media adjusted to pH 6.5 or 6.7 for various time points as indicated. Total RNA and proteins were extracted and subjected to RT-PCR or immunobloting, respectively. Primers used for PCR experiments are shown in Table S1. For immunoblot analyses total proteins (25 µg/lane) were resolved by SDS-PAGE and NHE2 protein levels were detected by NHE2 antibody. Subsequently the membranes were stripped and re-probed with actin antibody using standard protocols. Egr-1 expression (B) in the same cell extracts was also analyzed by hybridization to Egr-1 antibody (1∶600 dilution).
(TIF)

Table S1 Oligonucleotides used for RT- PCR and ChIP analyses.
(DOC)
