# Supplementary material for: Extracellular Acidosis Stimulates NHE2 Expression through Activation of Transcription Factor Egr-1 in the Intestinal Epithelial Cells
Source: PLoS One. 2013 Dec 23;8(12):e82023. doi: 10.1371/journal.pone.0082023 (PMC3871166; doi:10.1371/journal.pone.0082023)
Supplement: Table S1 — Oligonucleotides used for RT- PCR and ChIP analyses. (DOC) [file pone.0082023.s002.doc]

**Table S1. Oligonucleotides used for RT- PCR and ChIP analyses.**

**Primers Primer Sequences**

**NHE2 mRNA:**

F- #207 5’-ACTATTCGACCACTGGTGGAG-3’

R- #39 5’-ACTTATCATCCCAGTCTCTGCC-3’

**NHE2 mRNA:**

F- #137 5’-TTATTACGGCTGCCATTGTTGT-3’

R- #179 5’-TGTCTCTCCAAAAGTTGTG-3’

**NHE2 hnRNA:**

F- #137 5’-TTATTACGGCTGCCATTGTTGT-3’

R- int-5 5’-CCAGTCATAAGATTAGAGCGC-3’

**Egr-1 mRNA:**

F- Egr1 5’-CTTTCCTCACTCGCCCACCAT-3’

R- Egr1 5’-CAGCACCTTCTCGTTGTTCAG-3’

**ChIP:**

Forward -514 5’-TGGCTTTCCTAAACTTTCTGTACGTCCAGAGAATC-3’

Reverse -317 5’-CCCTCCTCCCCCGCCCCCCGCAGGCTCC-3’
